# DAB2IP Downregulation Enhances the Proliferation and Metastasis of Human Gastric Cancer Cells by Derepressing the ERK1/2 Pathway

**DOI:** 10.1155/2018/2968252

**Published:** 2018-03-21

**Authors:** Liang Sun, Yizhou Yao, Ting Lu, Zengfu Shang, Shenghua Zhan, Weiqiang Shi, Guofeng Pan, Xinguo Zhu, Songbing He

**Affiliations:** ^1^Department of General Surgery, The First Affiliated Hospital of Soochow University, Suzhou, Jiangsu 215006, China; ^2^Department of Gastroenterology, The First Affiliated Hospital of Soochow University, Suzhou, Jiangsu 215006, China; ^3^Department of Radiation Medicine, Medical College of Soochow University, Suzhou 215006, China; ^4^Department of Pathology, The First Affiliated Hospital of Soochow University, Suzhou, Jiangsu 215006, China

## Abstract

DAB2IP (DOC2/DAB2 interactive protein) is downregulated in several cancer types, and its downregulation is involved in tumor cell proliferation, apoptosis, metastasis, and epithelial-mesenchymal transition (EMT). We aimed to investigate the potential role of DAB2IP in the development and progression of gastric cancer. DAB2IP levels were analyzed in human gastric cancer and adjacent normal tissues by Western blots and immunohistochemistry. Potential roles of DAB2IP in regulating gastric cancer cell growth and metastasis were examined by genetic manipulation in vitro. The molecular signaling was determined to understand the mechanisms of observed DAB2IP effects. DAB2IP level is lower in gastric cancer tissues as compared to paired normal tissues. Knockdown of DAB2IP enhanced gastric cancer cell growth and metastasis in vitro and promoted EMT progress at both protein and mRNA levels. Silencing DAB2IP activated extracellular signal-regulated kinase 1/2 (ERK1/2) pathway, and the enhanced proliferation and migration ability induced by DAB2IP knockdown were reduced after incubation with U0126 in SGC7901 gastric cancer cells. Inhibition of DAB2IP enhances gastric cancer cell growth and metastasis through targeting the ERK1/2 signaling, indicating that it may serve as a potential target for treatment of gastric cancer.

## 1. Introduction

Gastric cancer (GC) is one of the most common malignancies and the second leading cause of cancer death worldwide [1, 2]. Though therapeutic strategies are continually improved, overall 5-year survival is still less than 30% because most gastric cancers are diagnosed at advanced or metastatic stages [3–5]. Therefore, it is urgent to explore specific and sensitive biomarkers for early diagnosis of gastric cancer, and identification of molecules suppressing metastasis of tumor cells may provide novel targets for therapy of gastric cancer.

DAB2IP (DOC2/DAB2 interactive protein), a member of the RAS-GTPase-activating protein (RAS-GAP) family [6], is downregulated in several cancer types, such as prostate cancer, bladder cancer, hepatocellular cancer, and colorectal cancer [7–10]. In cancers, downregulation of DAB2IP has been demonstrated to be involved in tumor cell proliferation, apoptosis, metastasis, and epithelial-mesenchymal transition (EMT) through several signaling pathways, including Ras-ERK, PI3K-Akt, ASK1-JNK, and NF-*κ*B [7, 11–13]. However, there are just two studies focusing on DAB2IP in gastric cancer [14, 15], which indicate that DAB2IP methylation is frequently present in gastrointestinal tumors and the resulting gene silencing plays an important role in gastrointestinal carcinogenesis. These data suggest that low expression of DAB2IP contributes to the development and progression of gastric cancer, but the precise function and internal mechanisms of DAB2IP in gastric cancer cell growth and metastasis remain elusive.

Herein, we detected the expression of DAB2IP in gastric tumor tissue specimens by Western blots and immunohistochemical (IHC) staining and investigated the effect of DAB2IP knockdown on gastric cancer cell proliferation and migration in vitro. We also examined its effect on the regulation of ERK1/2 signaling pathway, which strongly associates with cell growth, metastasis, and EMT in gastric cancer [16–18]. Our in vitro studies demonstrate that DAB2IP functions as a novel regulator for ERK1/2 signaling pathway to mediate gastric cancer cell growth and metastasis and reveal its potential implications for new approaches to gastric cancer therapy.

## 2. Materials and Methods

### 2.1. Human Gastric Cancer Tissues and Cell Lines

Human gastric cancer tissues and adjacent normal tissues were collected immediately after surgical resection at the First Affiliated Hospital of Soochow University (Suzhou, Jiangsu, China). The researches were supported by the Independent Ethics Committee (IEC) of the First Affiliated Hospital of Soochow University, and all patients provided written informed consent. The human gastric cancer cell lines were all purchased from the Cell Bank of the Chinese Academy of Sciences (Shanghai, China) and were cultured in Roswell Park Memorial Institute (RPMI) 1640 medium (Hyclone) containing 10% FBS (Gibco), 100 units/ml penicillin G sodium, and 100 *μ*g/ml streptomycin sulfate (Gibco). Both cells were maintained at 37°C in a humidified atmosphere containing 5% CO_2_.

### 2.2. Immunohistochemistry (IHC)

Surgical specimens were fixed in 10% formalin and embedded in paraffin. Briefly, the paraffin-embedded tissues were serially cut into 5 *μ*m sections and incubated with the polyclonal antibody recognizing human DAB2IP at 1 : 200 dilutions 4°C overnight. The proteins were visualized using a tissue staining kit (Zhongshan Biotechnology, Beijing, China), and staining scores were examined by two clinical pathologists. Five random regions were analyzed and the presence of brown-colored granules on the cytoplasm was taken as a positive signal. The staining was divided by color intensity into not colored, light yellow, brown, and tan and is recorded as 0, 1, 2, and 3, respectively. Positive cell rate of <25% was a score of 1, positive cell rate of 25–50% was a score of 2, positive cell rate of 51–75% was a score of 3, positive cell rate of >75% was a score of 4. An average intensity score of 4 or above was considered as high expression and a score below 4 as none or low expression.

### 2.3. Generation of Stable Cell Lines

AGS and SGC7901 cell lines that stably expressed DAB2IP-specific short hairpin RNA (shRNA) and scrambled shRNA control were constructed using a lentivirus vector-based shRNA technique. The human DAB2IP shRNA targets are 5′-GGG AUA GGC UAA GGA GUAATT-3′ (shDAB2IP#1) and 5′-CGC UGU AUG AGU CAG AUGATT-3′ (shDAB2IP#2). Oligonucleotides were constructed in lentiviral RNAi vector (GenePharma, Shanghai, China). After cotransfection of lentiviral packaging plasmids into 293 T cells, lentivirus-containing supernatant was obtained 48 h after transfection. AGS and SGC7901 cells were transduced with serial dilutions of lentiviral supernatant and selected by 4 *μ*g/ml puromycin for 2 weeks.

### 2.4. Protein Extraction and Western Blot Analysis

Whole protein extracts were lysed in ice-cold RIPA lysis buffer containing cocktails of protease and phosphatase inhibitors (Sigma) according to the manufacturer's protocol. Total proteins from each lysate were separated by SDS-PAGE and transferred onto PVDF membranes and then blocked with 5% nonfat milk for 1 hour. The membranes were then probed with the indicated primary antibodies at 4°C with gentle shaking overnight and incubated with horseradish peroxidase- (HRP-) conjugated secondary antibodies. Then, the proteins were visualized by chemiluminescence, and signals were quantified by Image J software. Antibodies used in this study are listed in Table 1.

### 2.5. RNA Extraction and Quantitative Real-Time Reverse Transcription PCR (QRT-PCR)

Total RNA was extracted from cells using TRIzol Reagent (Invitrogen, Life Technologies), and cDNA was synthesized from 2 *μ*g of RNA using the First Strand cDNA Synthesis Kit (Fermentas) according to the manufacturer's instructions. QRT-PCR was carried out using Power SYBR® Green PCR Master Mix (ABI, USA) on the 7500 real-time PCR system (ABI, life technology). 18 s was used as a loading control for each specific gene. The sequences for sense (S) and antisense (AS) primers are as follows: human-DAB2IP-S, 5′-CTGAGCGGGATAAGTGGATGG-3′, human-DAB2IP-AS, 5′-AAACATTGTCCGTCTTGAGCTT-3′, human-E-cadherin-S, 5′-CGGGAATGCAGTTGAGGATC-3′, human-E-cadherin-AS, 5′-AGGATGGTGTAAGCGATGGC-3′, human-Vimentin-S, 5′-GAGAACTTTGCCGTTGAAGC-3′, human-Vimentin-AS, 5′-GCTTCCTGTAGGTGGCAATC-3′, human-18 s-S, 5′-GTAACCCGTTGAACCCCATT-3′, human-18 s-AS, 5′-CCATCCAATCGGTAGTAGCG-3′. The PCR conditions consisted of 5 min at 95°C 1 cycle, 30 sec at 95°C, 30 sec at 55°C, 30 sec at 72°C, and 7 min at 72°C 40 cycles. The relative fold changes in mRNA expression were calculated using the 2^−∆∆CT^ method, where the average of ∆CT values for the amplicon of interest was normalized to that of 18 s and compared with the control specimens.

### 2.6. MTT Assay of Cell Viability

Cell viability was determined using an MTT assay kit (Amresco, USA). After transfection, 2000 cells were seeded in 96-well plates for 1 d, 2 d, 3 d, 4 d, and 5 d and then incubated with MTT solution-containing culture medium at 37°C for 4 hours. Then supernatants were removed, and formazan crystals were dissolved in 150 *μ*l DMSO. After gentle shaking for 10 minutes, the absorbance at 490 nm was measured by using a microplate reader.

### 2.7. Colony Formation Assay

1000 cells were placed in 6-well plates for 10 days and then fixed and stained with 0.1% crystal violet. The number of foci containing >100 cells was determined at 40x magnification using an optical microscope (Nikon), and the images were taken by a digital camera (Nikon).

### 2.8. Cell Migration Assay

Gastric cancer cell migration was assessed using the Transwell chambers (pore size, 8.0 *μ*m; Corning, New York, USA). The cells were resuspended in serum-free RPMI medium, then cell suspensions (200 *μ*l containing 50,000 cells) were seeded onto the filters in 24-well chambers; 750 *μ*l of medium containing 10% FBS was placed in the lower chambers as a chemoattractant. The cells were allowed to migrate for 24 h at 37°C. Cells remaining on the upper surface of the membrane were removed using a cotton swab. The filters were fixed with 4% paraformaldehyde, and the cells were stained with 0.1% crystal violet solution. The cells that had migrated from the upper to the lower side of the filter were counted in 5 randomly selected fields per sample.

### 2.9. Statistical Analysis

Data are presented as mean ± SEM. Statistical significance was analyzed using the Student *t*-test (unpaired, two-tailed) or one-way ANOVA. Pearson *χ*^2^ test was used to analyze the relationships between DAB2IP expression and clinicopathologic factors. A value of *P* < 0.05 was considered to indicate a statistically significant difference.

## 3. Results

### 3.1. Downregulation of DAB2IP in Human Gastric Cancer Correlates with Tumor Size, Lymph Node Metastasis, and TNM Stage

**Figure 1:**
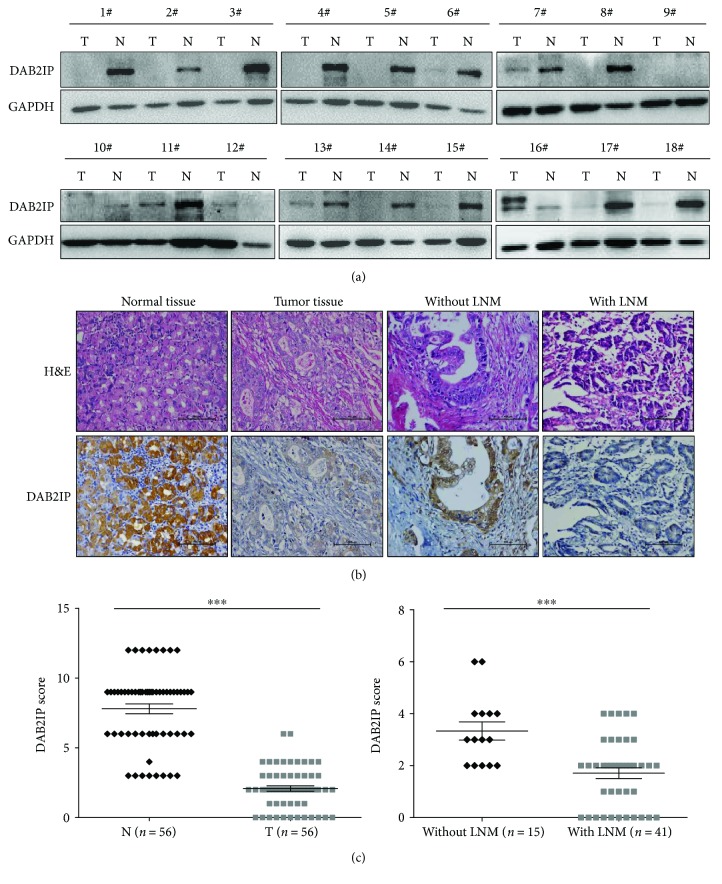
DAB2IP is downregulated in human gastric cancer. (a) Western blotting of DAB2IP protein in gastric cancer tumors. Eighteen randomly selected pairs of gastric cancer tumors (T) and matched normal tissues (N) are presented. GAPDH as a loading control. (b) Immunohistochemical staining of DAB2IP in representative carcinoma and the surrounding normal tissues of gastric cancer (magnification, ×200). (c) Scatter plot analysis of DAB2IP levels in 56 gastric cancer tumors (T) and paired normal tissues (N). Statistical significance was determined by a two-tailed, unpaired Student *t*-test. ^∗∗∗^*P* < 0.001.

To investigate the potential role of DAB2IP in the development of gastric cancer, we first used Western blotting to examine DAB2IP expression in human gastric cancer tissues and paired adjacent normal tissues. Strikingly, our results showed that abundant DAB2IP expression was observed in the matched paraneoplastic tissues of gastric cancer. On the contrary, the expression of DAB2IP was obviously lower in the primary gastric cancer tumors (Figure 1(a)). Immunohistochemical staining confirmed that the expression of DAB2IP is downregulated in tumor tissues and is further reduced in those with lymph node metastasis (Figures 1(b) and 1(c)). We next investigated the relationship between DAB2IP expression levels and the clinicopathologic status of patients with gastric cancer and found that reduced DAB2IP in gastric cancer cells was significantly correlated with tumor size, lymph node metastasis, and TNM stage (Table 2). These data suggest that reduced DAB2IP expression is associated with the development of gastric cancer.

### 3.2. Expression of DAB2IP in Human Gastric Cancer Cells and shRNA against DAB2IP Inhibits the mRNA and Protein Levels of DAB2IP Expression

**Figure 2:**
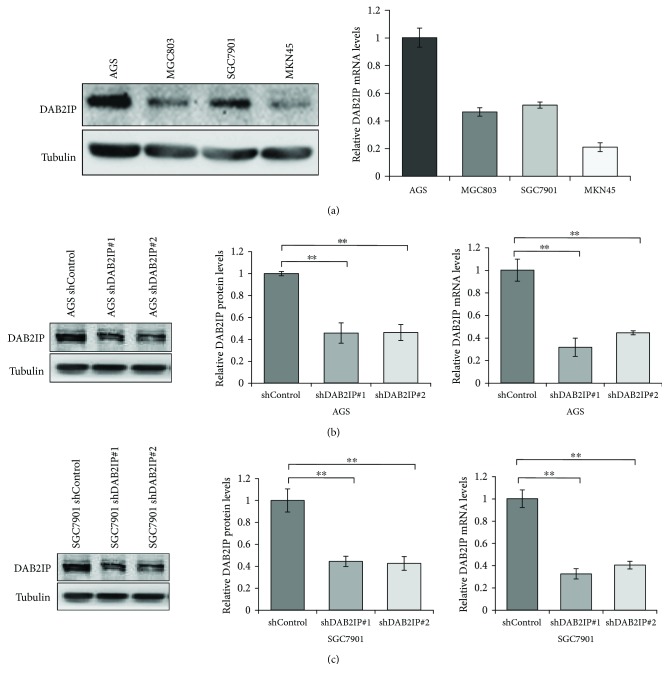
Expression of DAB2IP in human gastric cancer cells and the knockdown efficiency of DAB2IP. (a) DAB2IP protein and mRNA expression in four gastric cancer cell lines were detected by Western blot and QRT-PCR. The bar graphs represent the 18 s-normalized DAB2IP mRNA levels. Error bars represent SEM (*n* = 3). (b, c) The protein and mRNA levels of DAB2IP expression in wild-type cells (shControl) and in cells with stable knockdown of DAB2IP (shDAB2IP) were tested by Western blot and QRT-PCR in AGS (b) and SGC7901 (c) gastric cancer cells. Error bars represent SEM (*n* = 3). Statistical significance was determined by a two-tailed, unpaired Student *t*-test. ^∗∗^*P* < 0.01.

To evaluate the baseline expression levels of DAB2IP in a series of human gastric cancer cell lines, we detected the mRNA and protein expression of DAB2IP by QRT-PCR and Western blotting analysis, respectively. Our results evidenced that AGS cells and SGC7901 cells expressed a high level of DAB2IP at both mRNA and protein levels (Figure 2(a)). We then stably knocked down DAB2IP expression using a lentivirus vector-based shRNA technique in AGS and SGC7901 cell lines, and the knockdown efficiency was confirmed by Western blot and QRT-PCR analysis (Figures 2(b) and 2(c)).

### 3.3. DAB2IP Deficiency Promotes Gastric Cancer Cell Growth and Metastasis In Vitro

**Figure 3:**
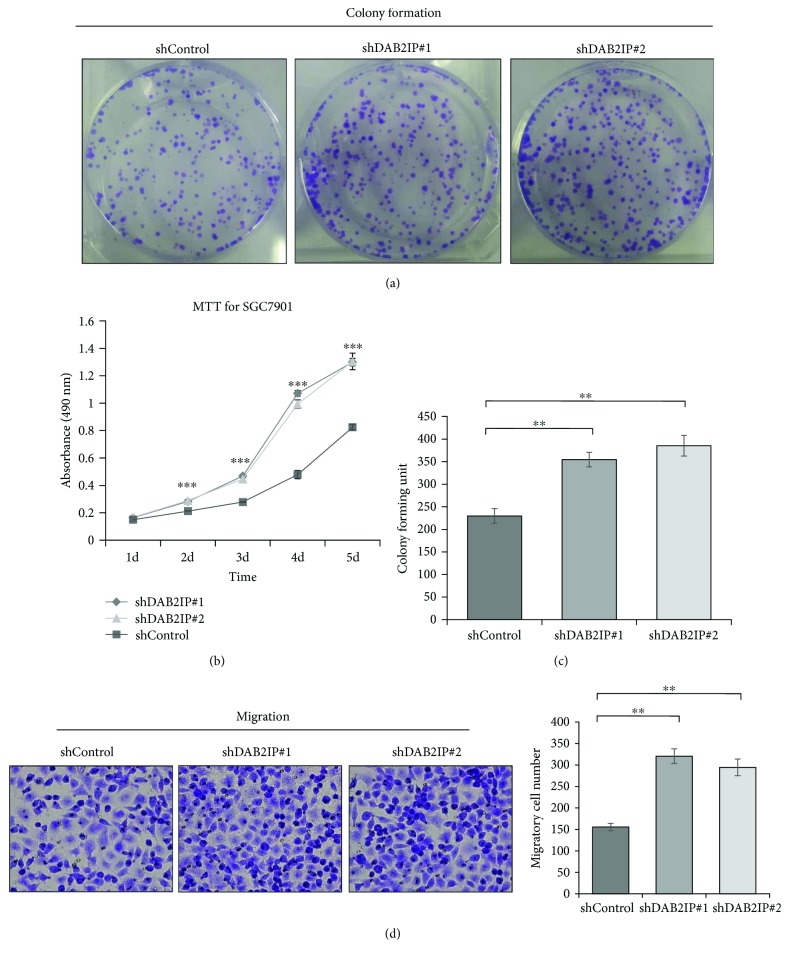
Knockdown of DAB2IP promotes gastric cancer cell growth and metastasis in vitro. (a, c) SGC7901 cells stably expressing the shRNA vector were maintained in culture media for 10 days and then fixed and stained with 0.1% crystal violet, and the colonies containing above 100 cells were counted manually. The representative photographs are presented (a; magnification, ×1), and the relative number of colonies was counted (c). The bands were quantified and presented as the mean ± SEM of three independent experiments. (b) Figures are curves of SGC7901 cell growth by MTT assays, presented as mean ± SEM (*n* = 5). (d) Migration assays were performed in wild-type cells (shControl) and in cells with stable knockdown of DAB2IP (shDAB2IP). Representative photographs are presented (left; magnification, ×200) and the relative number of migratory cells (right) was counted. The bands were quantified and presented as the mean ± SEM of three independent experiments. Statistical significance was determined by a two-tailed, unpaired Student *t*-test or one-way ANOVA. ^∗∗^*P* < 0.01; ^∗∗∗^*P* < 0.001.

Since DAB2IP expression is downregulated in gastric cancer tissues, we thus asked whether DAB2IP has a causal role in regulating gastric cancer cell phenotypes. Colony formation assays showed that the ability of SGC7901 cells to form foci was greatly enhanced when cells lacked DAB2IP (Figures 3(a) and 3(c)). In agreement with this result, MTT assays revealed that silencing DAB2IP in SGC7901 cells significantly enhanced the ability of cell proliferation (Figure 3(b)). In a transwell migration assay, DAB2IP-depleted SGC7901 cells migrated approximately twice as much as the control cells (Figure 3(d)). These results indicate that DAB2IP deficiency promotes gastric cancer cell growth and metastasis in vitro.

### 3.4. Silencing DAB2IP Expression Promotes EMT Progress at Both Protein and mRNA Levels in Gastric Cancer Cells

**Figure 4:**
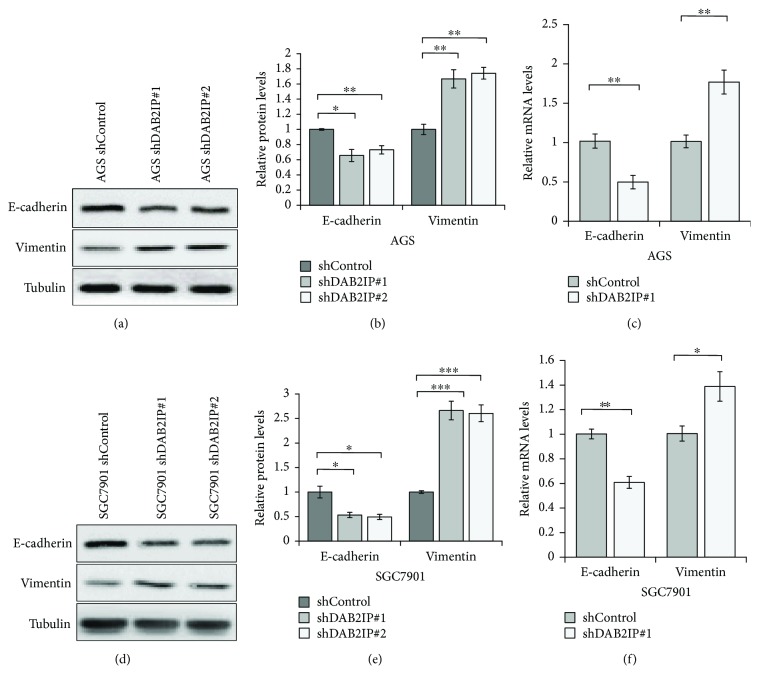
Downregulation of DAB2IP expression promotes EMT at both protein and mRNA levels in gastric cancer cells. (a) Western blotting analysis of the E-cadherin and vimentin expression in wild-type AGS cells (shControl) and in cells with stable knockdown of DAB2IP (shDAB2IP). Tubulin as a loading control. (b) The bands were quantified and presented as the mean ± SEM of three independent experiments. (c) QRT-PCR analysis of the E-cadherin and vimentin mRNA expression in wild-type AGS cells (shControl) and in cells with stable knockdown of DAB2IP (shDAB2IP). The bands were presented as the mean ± SEM (*n* = 3). (D) Western blotting analysis of the E-cadherin and vimentin expression in wild-type SGC7901 cells (shControl) and in cells with stable knockdown of DAB2IP (shDAB2IP). Tubulin as a loading control. (e) The bands were quantified and presented as the mean ± SEM of three independent experiments. (f) QRT-PCR analysis of the E-cadherin and vimentin mRNA expression in wild-type SGC7901 cells (shControl) and in cells with stable knockdown of DAB2IP (shDAB2IP). The bands were presented as the mean ± SEM (*n* = 3). Statistical significance was determined by a two-tailed, unpaired Student *t*-test. ^∗^*P* < 0.05; ^∗∗^*P* < 0.01; ^∗∗∗^*P* < 0.001.

Since DAB2IP is involved in gastric cancer metastasis, it is possible that DAB2IP may regulate EMT progress, which is an early event in the metastasis of cancer [19, 20]. To test this, the expression of the epithelial marker E-cadherin and the mesenchymal marker vimentin was analyzed using QRT-PCR and Western blotting assays. Our results revealed that silencing DAB2IP reduced E-cadherin expression but increased vimentin expression at both protein and mRNA levels (Figures 4(a), 4(b), and 4(c)), indicating that DAB2IP exerts a crucial role in modulating EMT in gastric cancer cells.

### 3.5. The Tumor-Promoting Effects Induced by DAB2IP Knockdown on Gastric Cancer Cells Are Mediated through the Activation of ERK1/2 Pathway

**Figure 5:**
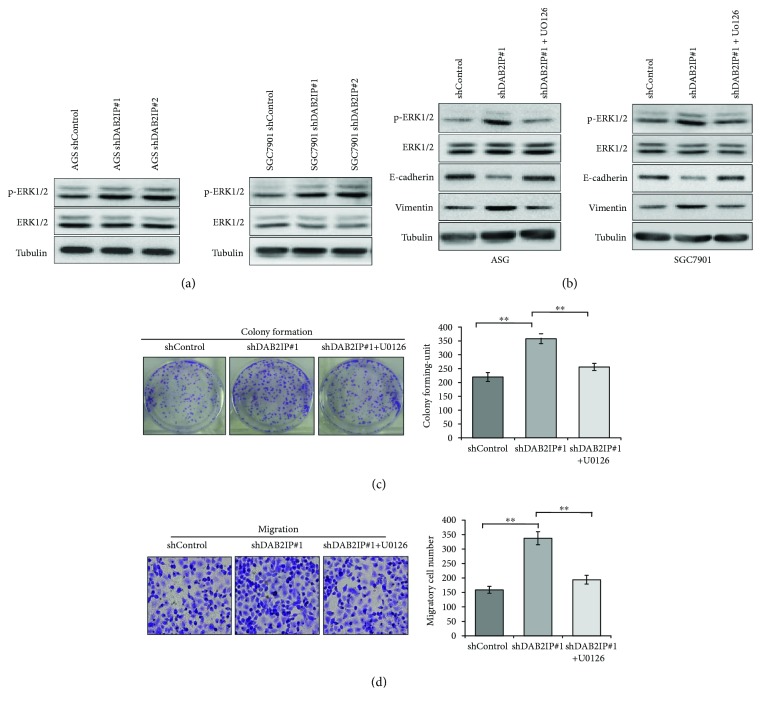
DAB2IP knockdown promotes gastric cancer cell growth and metastasis in a ERK1/2 signaling pathway manner. (a) Western blotting analysis of the p-ERK1/2 and ERK expression in wild-type cells (shControl) and in cells with stable knockdown of DAB2IP (shDAB2IP). Tubulin as a loading control. (b) Western blot was used to detect the level of p-ERK1/2, ERK1/2, E-cadherin, and vimentin in the presence or absence of U0126 (20 *μ*M). (c) Colony formation assays were performed in wild-type SGC7901 cells (shControl) and in cells with stable knockdown of DAB2IP (shDAB2IP) with or without U0126 (20 *μ*M). Representative photographs are presented (left; magnification, ×1), and the colonies containing above 100 cells were counted (right). The bands were quantified and presented as the mean ± SEM of three independent experiments. (d) Migration assays were performed in wild-type SGC7901 cells (shControl) and in cells with stable knockdown of DAB2IP (shDAB2IP) with or without U0126 (20 *μ*M). Representative photographs are presented (left; magnification, ×200), and the relative number of migratory cells (right) were counted. The bands were quantified and presented as the mean ± SEM of three independent experiments. Statistical significance was determined by a two-tailed, unpaired Student *t*-test. ^∗∗^*P* < 0.01.

Given the evidence that ERK1/2 signaling pathway was identified as one of the most significantly altered pathway when DAB2IP was knockdown in cancers [13] and the ERK1/2 signaling pathway plays a central role in the process of tumor cell growth and metastasis [21–23], we sought to test whether ERK1/2 pathway is implicated in the DAB2IP-mediated gastric cancer cell growth and metastasis. As expected, we found that knockdown of DAB2IP obviously augmented the level of phosphorylated ERK1/2 (p-ERK1/2) in both AGS and SGC7901 gastric cancer cells (Figure 5(a)). To further confirm that DAB2IP regulates cell growth and EMT via ERK1/2 pathway, we treated DAB2IP-deficient cells with U0126, a highly selective inhibitor of both MEK1 and MEK2 (a type of MAPK/ERK kinase). As shown in Figure 5(b), EMT induced by DAB2IP knockdown was significantly reversed after treatment with U0126 in both AGS and SGC7901 gastric cancer cells. In agreement with this result, colony formation assays and migration assays showed that the enhanced proliferation and migration ability induced by blockade of DAB2IP were significantly reduced after incubation with U0126 in SGC7901 gastric cancer cells (Figures 5(c) and 5(d)). These data suggest that DAB2IP modulates the growth and metastasis of gastric cancer cells via regulation of ERK1/2 signaling pathway.

## 4. Discussion

Recently, DAB2IP was identified as a DOC-2/DAB2 interactive protein with the growth-inhibitory effect in prostate cancer [24]. Since then, the functions of DAB2IP have extended to regulating cell proliferation, metastasis, EMT, cancer stem cell phenotype, radiation, and chemotherapy resistance [11–13, 24–28]. DAB2IP is a member of the RAS-GTPase-activating protein (RAS-GAP) family [6] and is downregulated in several cancer types, including prostate cancer, bladder cancer, hepatocellular cancer, and colorectal cancer [7–10], suggesting that DAB2IP has emerged as an attractive target for cancer therapy.

However, studies focusing upon DAB2IP in gastric cancer are scanty. Previous studies [14, 15] have provided evidence that DAB2IP methylation is frequently present in gastrointestinal tumors, and the resulting gene silencing plays an important role in gastrointestinal carcinogenesis, indicating that downregulation of DAB2IP contributes to the development and progression of gastric cancer. However, the functional role of DAB2IP and the concrete molecular mechanisms in gastric cancer remain unclear and need to be explored.

Emerging literature suggests that EMT plays a crucial role in tumor cell metastasis and invasion, which is accompanied by upregulation of mesenchymal-associated genes such as vimentin and downregulation of epithelial-associated markers such as E-cadherin [19, 20]. Several types of signaling and molecules are involved in the regulation of EMT, such as transforming growth factor *β* (TGF-*β*) [28] Hedgehog [29], Notch [30], and MAPK/ERK [21–23].

In the present study, we demonstrated for the first time that the expression of DAB2IP in gastric cancer tissue was downregulated compared to the adjacent normal tissues. We also provided the first evidence that knockdown of DAB2IP enhanced proliferation and migration of gastric cancer cells. In view of the important role of EMT in tumor metastasis during tumor progression [19, 20], we explored the effect of DAB2IP on EMT progress and revealed that DAB2IP can modulate the expression of EMT marker E-cadherin and vimentin in AGS and SGC7901 gastric cancer cells.

Considering that DAB2IP plays a critical role in regulating the activation of ERK1/2 pathway in colorectal cancer [13, 31] and activation of ERK1/2 signaling pathway contributes to tumor cell growth and metastasis [21–23], we asked whether DAB2IP modulates the growth and metastasis of gastric cancer cells via regulating ERK1/2 signaling pathway. In accordance with other studies [13, 31], blockade of DAB2IP enhanced the phosphorylation of ERK1/2 in gastric cancer cells (Figure 5(a)) and the enhanced proliferation and migration ability induced by DAB2IP knockdown was remarkably reduced after incubation with U0126 in SGC7901 gastric cancer cells (Figures 5(c) and 5(d)). Furthermore, EMT induced by DAB2IP knockdown was reversed after treatment with U0126 in both AGS and SGC7901 gastric cancer cells (Figure 5(b)). These data indicate that DAB2IP regulates the growth, metastasis, and EMT of gastric cancer cells via regulation of ERK1/2 signaling pathway.

However, there are some limitations in our study. Firstly, we did not investigate the effect of depletion of DAB2IP on gastric cancer tumorigenesis and metastasis in vivo. Secondly, DAB2IP overexpression in gastric cancer cells was not carried out because we failed to construct the expressing vector due to the long coding domain of DAB2IP gene. Thus, more research should be undertaken to further explore the function of DAB2IP in gastric cancer.

In conclusion, our present study not only indicates that DAB2IP is downregulated in gastric cancer tissues compared to the adjacent normal tissues but also emphasizes the effect of DAB2IP knockdown on gastric cancer cell growth and metastasis in vitro. Moreover, we reveal a critical mechanism for DAB2IP in the regulation of gastric cancer tumorigenesis and metastasis through its participation in EKR1/2 signaling pathway. This may highlight a new entry point for treating gastric cancer by targeting the DAB2IP/ERK1/2 signaling axis.

## Figures and Tables

**Table 1:** Antibody information.

Name	Company	Catalog number	Antibody concentration
DAB2IP	Abcam	ab87811	1 : 1000 or 1 : 200
E-cadherin	Cell Signaling Technology	3195	1 : 500
Vimentin	Cell Signaling Technology	5741	1 : 500
p-ERK1/2	Cell Signaling Technology	4370	1 : 1000
ERK1/2	Cell Signaling Technology	4695	1 : 1000
GAPDH	Beyotime	AG019	1 : 2000
*α*-tubulin	Sigma-Aldrich	T6199	1 : 2000

**Table 2:** Relationship between DAB2IP expression and clinic-pathological features in patients with gastric cancer.

Clinic parameters	Total	DAB2IP expression		*P* value
		None or low	High	
*Age*				
<65	26	20	6	0.5471
≥65	30	25	5	
*Gender*				
Male	40	31	9	0.3948
Female	16	14	2	
*Tumor size*				
<5 cm	27	18	9	0.0128^∗^
≥5 cm	29	27	2	
*Tumor location*				
U&M	34	30	4	0.0651
L	22	15	7	
*Histological differentiation*				
Well or moderate	30	24	6	0.9424
Poor	26	21	5	
*Depth of invasion*				
T1-2	10	6	4	0.0738
T3-4	46	39	7	
*Lymph node metastasis*				
Yes	41	36	5	0.0204^∗^
No	15	9	6	
*TNM stage*				
I/II	17	10	7	0.0074^∗∗^
III/IV	39	35	4	

^∗^ *P* < 0.05; ^∗∗^*P* < 0.01.
