# Erratum to “DAB2IP Downregulation Enhances the Proliferation and Metastasis of Human Gastric Cancer Cells by Derepressing the ERK1/2 Pathway”

**DOI:** 10.1155/2020/7419534

**Published:** 2020-04-01

**Authors:** Liang Sun, Yizhou Yao, Ting Lu, Zengfu Shang, Shenghua Zhan, Weiqiang Shi, Guofeng Pan, Xinguo Zhu, Songbing He

**Affiliations:** ^1^Department of General Surgery, The First Affiliated Hospital of Soochow University, Suzhou, Jiangsu 215006, China; ^2^Department of Gastroenterology, The First Affiliated Hospital of Soochow University, Suzhou, Jiangsu 215006, China; ^3^Department of Radiation Medicine, Medical College of Soochow University, Suzhou 215006, China; ^4^Department of Pathology, The First Affiliated Hospital of Soochow University, Suzhou, Jiangsu 215006, China

In the article titled “DAB2IP Downregulation Enhances the Proliferation and Metastasis of Human Gastric Cancer Cells by Derepressing the ERK1/2 Pathway” [1], there was an error in Figure 2, where the Western blots in Figures 2(b) and 2(c) were duplicated, due to a production error. The correct figure is as follows.

## Figures and Tables

**Figure 1:**
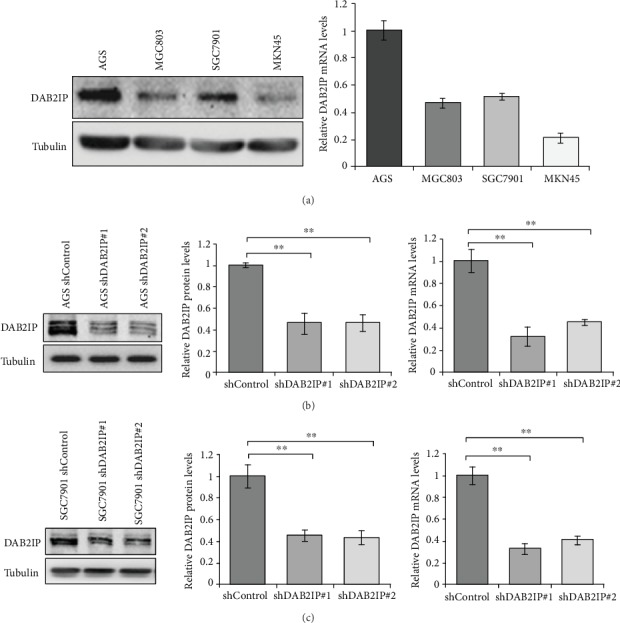
Expression of DAB2IP in human gastric cancer cells and the knockdown efficiency of DAB2IP. (a) DAB2IP protein and mRNA expression in four gastric cancer cell lines were detected by Western blot and QRT-PCR. The bar graphs represent the 18 s-normalized DAB2IP mRNA levels. Error bars represent SEM (*n* = 3). (b, c) The protein and mRNA levels of DAB2IP expression in wild-type cells (shControl) and in cells with stable knockdown of DAB2IP (shDAB2IP) were tested by Western blot and QRT-PCR in (b) AGS and (c) SGC7901 gastric cancer cells. Error bars represent SEM (*n* = 3). Statistical significance was determined by a two-tailed, unpaired Student's *t*-test. ^∗∗^*P* < 0.01.
